# Mercury exposure and risk of cardiovascular disease: a nested case-control study in the PREDIMED (PREvention with MEDiterranean Diet) study

**DOI:** 10.1186/s12872-016-0435-8

**Published:** 2017-01-05

**Authors:** Mary K. Downer, Miguel A. Martínez-González, Alfredo Gea, Meir Stampfer, Julia Warnberg, Miguel Ruiz-Canela, Jordi Salas-Salvadó, Dolores Corella, Emilio Ros, Montse Fitó, Ramon Estruch, Fernando Arós, Miquel Fiol, José Lapetra, Lluís Serra-Majem, Monica Bullo, Jose V. Sorli, Miguel A. Muñoz, Antonio García-Rodriguez, Mario Gutierrez-Bedmar, Enrique Gómez-Gracia, PREDIMED Study Investigators

**Affiliations:** 1 Harvard T.H. Chan School of Public Health, Boston, USA; 2 Human Nutrition Unit, Faculty of Medicine and Health Sciences, IISPV, Rovira i Virgili University, Reus, Spain; 3 CIBERobn Physiopathology of Obesity and Nutrition, Institute of Health Carlos III, Madrid, Spain; 4 Department of Preventive Medicine and Public Health, University of Navarra, Pamplona, Spain; 5 Department of Preventive Medicine, University of Valencia, Valencia, Spain; 6 Lipid Clinic, Endocrinology and Nutrition Service, IDIBAPS, Hospital Clinic, University of Barcelona, Barcelona, Spain; 7 Cardiovascular Risk and Nutrition (Regicor Study Group), Hospital del Mar Medical Research Institute (IMIM), Barcelona, Spain; 8 Department of Internal Medicine, August Pi i Sunyer Institute of Biomedical Research (IDIBAPS), Hospital Clinic, University of Barcelona, Barcelona, Spain; 9 Department of Cardiology, University Hospital Araba, Vitoria, Spain; 10 Department of Preventive Medicine, University of Malaga, Malaga, Spain; 11 Institute of Health Sciences, University of Balearic Islands and Son Espases Hospital, Palma de Mallorca, Spain; 12 Department of Family Medicine, Primary Care Division of Sevilla, San Pablo Health Center, Sevilla, Spain; 13 Department of Clinical Sciences, University of Las Palmas de Gran Canaria, Las Palmas, Spain; 14 IdiSNA, Navarra Institute for Health Research, Pamplona, Spain; 15 Campus Teatinos, Facultad de Medicina, 29071 Malaga, Spain

**Keywords:** Mercury, Fish, Cardiovascular disease, Mediterranean diet, PREDIMED, Toenail biomarker

## Abstract

**Background:**

Substantial evidence suggests that consuming 1–2 servings of fish per week, particularly oily fish (e.g., salmon, herring, sardines) is beneficial for cardiovascular health due to its high n-3 polyunsaturated fatty acid content. However, there is some concern that the mercury content in fish may increase cardiovascular disease risk, but this relationship remains unclear.

**Methods:**

The PREDIMED trial included 7477 participants who were at high risk for cardiovascular disease at baseline. In this study, we evaluated associations between mercury exposure, fish consumption and cardiovascular disease. We randomly selected 147 of the 288 cases diagnosed with cardiovascular disease during follow-up and matched them on age and sex to 267 controls. Instrumental neutron activation analysis was used to assess toenail mercury concentration. In-person interviews, medical record reviews and validated questionnaires were used to assess fish consumption and other covariates. Information was collected at baseline and updated yearly during follow-up. We used conditional logistic regression to evaluate associations in the total nested case-control study, and unconditional logistic regression for population subsets.

**Results:**

Mean (±SD) toenail mercury concentrations (μg per gram) did not significantly differ between cases (0.63 (±0.53)) and controls (0.67 (±0.49)). Mercury concentration was not associated with cardiovascular disease in any analysis, and neither was fish consumption or n-3 fatty acids. The fully-adjusted relative risks for the highest versus lowest quartile of mercury concentration were 0.71 (95% Confidence Interval [CI], 0.34, 1.14; p_trend_ = 0.37) for the nested case-control study, 0.74 (95% CI, 0.32, 1.76; p_trend_ = 0.43) within the Mediterranean diet intervention group, and 0.50 (95% CI, 0.13, 1.96; p_trend_ = 0.41) within the control arm of the trial. Associations remained null when mercury was jointly assessed with fish consumption at baseline and during follow-up. Results were similar in different sensitivity analyses.

**Conclusions:**

We found no evidence that mercury exposure from regular fish consumption increases cardiovascular disease risk in a population of Spanish adults with high cardiovascular disease risk and high fish consumption. This implies that the mercury content in fish does not detract from the already established cardiovascular benefits of fish consumption.

**Trial registration:**

ISRCTN35739639.

**Electronic supplementary material:**

The online version of this article (doi:10.1186/s12872-016-0435-8) contains supplementary material, which is available to authorized users.

## Background

Overwhelming evidence demonstrates that even modest fish consumption (1–2 servings per week) has extensive health benefits, particularly for cardiovascular health. A recent systematic review reported that this relatively low level of fish consumption was associated with a 36% decreased risk of fatal coronary heart disease (CHD) and a 17% decreased risk of total mortality compared to <1 serving/week of fish consumption. Health benefits of modest but regular fish consumption occur within only months, but greater benefits occur with longer duration of intake. Oily fish (e.g., salmon, herring, sardines) may be especially beneficial, as they are relatively higher in two long-chain n-3 polyunsaturated fatty acids (n-3 PUFAs), eicosapentaenoic acid (EPA) and docosahexaenoic acid (DHA). EPA and DHA have been identified as the active constituents for cardiovascular benefits [1]. Furthermore, in many western societies it is likely that fish consumption displaces and thus reduces consumption of red and processed meat, and this reduction is associated with many health benefits [2–10].

Despite these health benefits of fish intake, there is concern about the potential adverse effects of mercury from fish; fish is a major source of mercury [11], which, at extremely high levels, has been shown to result in serious health complications including cardiovascular disease [1]. Several studies investigating the relationship between fish intake, mercury exposure and cardiovascular disease in adults have generated inconsistent results [12–19]. As a result, government agencies and health organizations have identified mercury exposure from fish consumption as an area needing further investigation [1, 20–23]. The present study seeks to clarify whether current Mediterranean diet recommendations of fish consumption (1–2 servings of fish per week), elevate mercury exposure to levels that increase risk of cardiovascular disease.

Extensive evidence supports that toenail concentration of methylmercury is an excellent biomarker for long-term mercury exposure [24–29]. A nested case-control study in the United States of 3427 cardiovascular disease cases and 3427 matched controls assessed mercury exposure by measuring toenail mercury concentration. They found no adverse effects of mercury exposure on coronary heart disease, stroke or total cardiovascular disease [11]. Another analysis using the same data found no adverse effects of mercury exposure on hypertension. This was true for levels 2.5-fold higher than the U.S. Environmental Protection Agency reference dose [30].

A 2012 review of mercury exposure and human health concluded that there is no clear relationship between mercury exposure and cardiovascular disease [31]. Thus, further evidence is needed before altering any policies or recommendations regarding mercury exposure through fish intake. We prospectively analyzed the associations between mercury exposure through fish intake and cardiovascular disease in PREDIMED, a large clinical trial of nutritional intervention for the primary prevention of cardiovascular disease. Our study population has unique characteristics for studying this association because our participants are at high risk of cardiovascular disease and they tend to consume large amounts of fish, and thus likely have higher levels of mercury exposure through fish consumption; per capita fish consumption in Spain is approximately twice the average of European countries [32], and fish consumption was even higher among PREDIMED participants randomized to the Mediterranean Diet.

## Methods

### Study design

The design and methods of the PREDIMED trial have been described previously [33, 34]. The PREDIMED trial was a randomized, controlled, cardiovascular disease prevention trial based in 11 centers throughout Spain [35]. Institutional Review Boards at all participating centers approved the study protocol.

Eligible participants included men (55–80 year) and women (60–80 year) at high risk for developing cardiovascular disease at enrollment, but had never been diagnosed with cardiovascular disease. High risk was defined as having type 2 diabetes mellitus or at least three of the following major risk factors: current smoking, hypertension, elevated low-density lipoprotein cholesterol levels, overweight or obesity or a familial history of premature coronary heart disease. 7447 participants were recruited between 2003 and 2009. After providing written informed consent, they were randomized to either a traditional Mediterranean diet supplemented with either extra virgin olive oil or tree nuts, or a control (low-fat) diet. The primary cardiovascular disease endpoint was defined as non-fatal acute myocardial infarction, non-fatal stroke or cardiovascular death. Hojiblanca and Patrimonio Communal Olivarero donated extra-virgin olive oil; the California Walnut Commission donated walnuts; Borges donated almonds; La Morella Nuts donated hazelnuts. Sponsors played no role in the design, analysis, interpretation or manuscript writing.

To evaluate the association between fish consumption, mercury exposure and cardiovascular disease, we designed a nested case-control study within the framework of the PREDIMED trial.

### Cases and controls

7,232 (97.1%) of PREDIMED participants provided toenail clippings at baseline, within 6 months of randomization. Among the PREDIMED participants who provided toenail samples, we randomly selected 147 of the 288 cases of incident cardiovascular disease (according to the definition of the primary end-point of the trial) during subsequent follow-up. The median follow-up period from time of toenail sampling to time of incident cardiovascular disease was 4.8 years (interquartile range 3.0–5.8 years). Outcomes were identified through repeated contacts with participants, contacts with family physicians, annual medical record review and consultation of the National Death Index. The End-Point Adjudication Committee was blinded to the randomized intervention group and every exposure, and ascertained outcomes. Only confirmed outcomes (non-fatal acute myocardial infarction, non-fatal stroke and cardiovascular death) were included as a case. Cases were randomly matched on age (within 2 years) and sex to 267 controls who were free from cardiovascular disease before December 2010 and had provided toenail samples at baseline. Most cases were matched to two controls, but 23 cases were matched to only one control.

### Measurement of exposure

The concentration of total methylmercury in the stored toenails was assessed using instrumental neutron activation analysis (INAA) at the Interfaculty Reactor Institute at Delft University of Technology in Delft, Netherlands. Supplemental materials provide details of analytic methods and validation of these methods [36].

Specifics of the dietary intervention are provided in detail elsewhere [37]. Registered dietitians conducted one-on-one in-person interviews to collect participants’ dietary information at baseline. We administered a validated 137-item food frequency questionnaire (FFQ) at baseline and yearly thereafter during the follow-up [38]. Participants reported how often, on average over the last year, they consumed each food item (never or almost never, 1–3 times per month, 1 time per week, 2–4 times per week, 5–6 times per week, 1 time per day, 2–3 times per day, 4–6 times per day and >6 times per day). Fish items include white fish (grouper, flounder, sea bream, hake, whiting; 1 serving), dark-meat fish (sardines, tuna, bonito, mackerel, salmon; 1 serving or 130 g); salted fish (cod or salted fish product; 1 serving or 60 g); oysters, clams, mussels (6 units); squid, octopus, cuttlefish (1 serving or 200 g); shellfish (shrimp, prawns, crayfish, etc.; 4–5 pieces or 200 g); natural canned seafood (sardines, anchovies, bonito, tuna; 1 small can or half normal can or 50 g); seafood in oil (sardines, anchovies, bonito, tuna; 1 small can or half normal can or 50 g). We used this information to calculate average fish consumption (in grams per day).

### Covariate assessment

Covariates were measured at baseline and yearly over follow-up. We reviewed medical records and used standardized validated protocols [39] to collect information on sociodemographic, lifestyle, health, family history and medical diagnoses. A validated Spanish version of the Minnesota Leisure Time Physical Activity Questionnaire [40] was used to evaluate physical activity. Trained nurses measured weight and height using standardized procedures, and blood pressure using a validated semiautomatic oscillometer in triplicate (Omron HEM_705CP). Yearly glucose tests identified new cases of diabetes. We developed and validated a 14-item Mediterranean diet adherence tool [41] to repeatedly assess adherence at baseline and during the intervention period. We used the validated FFQ described above to calculate total alcohol and energy intake. Primary care doctors assessed participants for hypercholestaerolemia, hypertension and type 2 diabetes diagnoses.

### Statistical analysis

To examine the association of toenail mercury concentration with cardiovascular disease in the nested case-control study’s total population, we used multivariate-adjusted conditional logistic regression, matching on age and sex. For separate analyses of the intervention groups or control group, we used multivariate-adjusted unconditional logistic regression, adjusting for matching factors. Mercury concentrations (μg/g) were categorized into quartiles based on the distribution among controls. To test for trend, we used the median value of the appropriate quartile of mercury and treated it as a continuous variable.

We again used multivariate-adjusted conditional logistic regression to evaluate mercury exposure in conjunction with fish consumption in the total nested case-control study. Baseline fish consumption was dichotomized into low and high groups according to the PREDIMED 14-item Mediterranean diet adherence tool (high: ≥3 servings/week) [41]. Mercury exposure was dichotomized as above or below the median value. We then created four exposure categories (low mercury/low fish, low mercury/high fish, high mercury/low fish, high mercury/high fish), which we included as indicator variables. The same conditional logistic regression methods were used to evaluate mercury exposure and increase in fish consumption in the nested case-control study. We cross-classified increase in fish consumption from baseline to three years of follow-up (increased or not increased) with baseline toenail mercury concentration (above or below median).

We also used multivariate-adjusted unconditional logistic regression to perform the following sensitivity analyses: 1) excluding diagnosed with cardiovascular disease within one year of baseline; 2) excluding those diagnosed over five years after toenail collection (baseline); 3) excluding participants above the 90th percentile of toenail selenium concentration; 4) stratifying on baseline fish intake (high/low), 5) restricting to participants at very high risk of cardiovascular disease (>70 years old with diabetes and hypertension at baseline); and 6) stratifying on baseline aspirin use (yes/no).

To control for potential confounders, we included in all models those variables based on clinical relevance and previous causal knowledge, as listed in the table footnotes. To evaluate the associations of fish and intake and mercury levels across the distribution of mercury exposure, we constructed restricted cubic splines.

All p-values are two-tailed. Values less than 0.05 are considered statistically significant. We performed all statistical analyses using Stata 12.0.

## Results

### Study population

Table 1 shows baseline characteristics for cases and controls. The mean (±SD) age was 69 years, and approximately 41% of the population was female. There was no significant difference in toenail mercury levels between cases and controls: the mean (±SD) level was 0.63 (±0.53) μg per gram for cases and 0.67 (±0.49) μg per gram for controls. Although they are measured on different scales, toenail mercury levels represent a well-established mercury biomarker which is closely correlated with blood levels (r = 0.78). For reference, 1 μg per gram of toenail mercury concentration corresponds to approximately 20 μg per liter of blood mercury concentration [42]. Levels of fish and seafood consumption, n-3 fatty acids and all other relevant dietary factors were similar. Statistically significant differences were observed only for history of type 2 diabetes (higher among cases) and Mediterranean diet adherence (higher among controls). This difference was expected, as both history of type 2 diabetes and poor adherence to the Mediterranean diet are risk factors for cardiovascular disease [43, 44].

**Table 1:** Baseline characteristics of case participants with incident cardiovascular disease and of controls in the PREDIMED

Characteristic	Case participants	Control participants	*p* value
*n*	147	271	
Age	69.2 (6.4)	68.6 (6.1)	Matching Factor
Female sex (%)	40.3	40.5	Matching Factor
PREDIMED trial arm (%)			0.03
Mediterranean diet + EVOO	37.4	37.6	
Mediterranean diet + nuts	24.5	35.1	
Smoking status (%)			0.39
Current smoker	19.7	15.1	
Former smoker	34.0	32.8	
Hypercholesterolaemia (%)	57.1	65.3	0.10
Hypertension (%)	80.3	77.5	0.51
Type 2 diabetes (%)	61.2	49.1	0.02
Family history of CHD (%)	19.7	15.5	0.27
Body mass index (kg/m^2^)	29.7 (3.6)	29.7 (3.5)	0.94
Physical activity (METs-min/d)	250.2 (211.1)	279.0 (272.5)	0.27
Alcohol (g/d)	10.1 (16.8)	12.7 (17.9)	0.15
Toenail mercury (μg/g)	0.63 (0.53)	0.67 (0.49)	0.42
Dietary intake			
Fish and seafood (g/d)	92.4 (46.0)	93.5 (44.1)	0.80
n-3 Fatty acids (EPA & DHA) (g/d)	0.71 (0.46)	0.75 (0.44)	0.34
n-3 Fatty acids (other)	1.41 (0.65)	1.44 (0.65)	0.72
Total energy intake (kcal/d)	2340 (645)	2363 (599)	0.71
Total Fat (%E)	39.5 (6.5)	39.7 (6.6)	0.70
Monounsaturated Fat (%E)	19.6 (4.4)	20.0 (4.6)	0.47
Polyunsaturated Fat (%E)	6.2 (2.2)	6.2 (1.8)	0.92
Saturated Fat (%E)	10.4 (2.3)	10.1 (2.2)	0.29
Protein (%E)	16.2 (2.9)	15.8 (2.7)	0.16
Cholesterol (mg/d)	363.0 (122.2)	366.5 (130.9)	0.78
Fiber (g/d)	24.9 (10.3)	25.1 (7.9)	0.78
Mediterranean diet adherence (0–14)	8.2 (2.0)	8.8 (1.8)	0.001
Fish item in Mediterranean screener (%)	52.1	51.7	0.94

Means (standard deviations) unless otherwise stated

*EVOO* extra-virgin olive oil, *CHD* coronary heart disease, *METs* metabolic equivalents, *EPA* eicosapentanoic acid, *DHA* docosahexanoic acid, *%E* percent of total energy intake, *OR* odds ratio, *CI* confidence Interval, *Hg* mercury

### Mercury exposure and risk of cardiovascular disease

The fully-adjusted relative risk (RR) for the highest versus lowest quartile of toenail mercury (Table 2) was 0.71 (95% Confidence Interval [CI], 0.34, 1.14; p_trend_ = 0.37) for the nested case-control study. When we stratified by the three randomized arms of the PREDIMED trial, the RRs were 0.74 (95% CI, 0.32, 1.76; p_trend_ = 0.43) within the Mediterranean diet intervention groups, and 0.50 (95% CI, 0.13, 1.96; p_trend_ = 0.41) within the control arm of the trial.

**Table 2:** Relative risk of cardiovascular disease, according to quartiles of toenail mercury, among case participants and matched controls in the PREDIMED trial

Variable		Sex-specific quartiles of toenail mercury				
	number of cases	Q1	Q2	Q3	Q4	p for trend
Mean mercury (μg/g)		0.25 (0.08)	0.43 (0.05)	0.65 (0.10)	1.30 (0.62)	
Total sample^a^						
Cases/matched controls	147/267	44/60	39/66	33/72	31/69	
Matched OR (95% CI)		1 (ref.)	0.93 (0.51, 1.70)	0.76 (0.42, 1.39)	0.60 (0.32, 1.13)	0.09
Matched OR^b^ (95% CI)		1 (ref.)	0.91 (0.48, 1.71)	0.84 (0.44, 1.59)	0.56 (0.28, 1.12)	0.09
Matched OR^c^ (95% CI)		1 (ref.)	0.86 (0.43, 1.72)	0.85 (0.43, 1.72)	0.66 (0.31, 1.41)	0.11
Mediterranean diet intervention^d^						
Cases/controls	91/197	23/39	25/49	20/55	23/54	
Adjusted OR (95% CI)		1 (ref.)	0.99 (0.46–2.12)	0.66 (0.30, 1.46)	0.72 (0.33, 1.55)	0.32
Adjusted OR^e^ (95% CI)		1 (ref.)	0.96 (0.43–2.14)	0.71 (0.30, 1.64)	0.66 (0.29–1.49)	0.27
Adjusted OR^f^ (95% CI)		1 (ref.)	1.00 (0.43, 2.32)	0.68 (0.28, 1.63)	0.74 (0.31, 1.75)	0.67
Control (low-fat) group^d^						
Cases/controls	56/74	21/22	14/17	13/18	8/18	
Adjusted OR (95% CI)		1 (ref.)	0.97 (0.35, 2.64)	0.78 (0.28, 2.17)	0.40 (0.12, 1.34)	0.13
Adjusted OR^e^ (95% CI)		1 (ref.)	1.08 (0.36, 3.23)	0.99 (0.33, 2.98)	0.44 (0.12, 1.64)	0.25
Adjusted OR^f^ (95% CI)		1 (ref.)	1.11 (0.34, 3.64)	1.06 (0.34, 3.38)	0.63 (0.15, 2.61)	0.75

^a^Models are from conditional logistic regression analyses with matching factors sex and age and adjusted for PREDIMED center

^b^Additionally adjusted for age, month of toenail collection, smoking, hypertension, hypercholestaerolemia, diabetes and family history of premature coronary heart disease

^c^Additionally adjusted for intervention group, baseline adherence to the Mediterranean diet, body mass index, physical activity (quartiles), alcohol intake (<5/10 gr, moderate consumption, >25/50 g in women/men), fish intake (continuous g/d) and non-fish consumption of n-3 fatty acids (continuous g/d)

^d^Models are from multivariate-adjusted logistic regression, adjusted for sex, age and PREDIMED center

^e^Additionally adjusted for month of toenail collection, smoking, hypertension, hypercholestaerolemia, diabetes and family history of premature coronary heart disease

^f^Additionally adjusted for intervention group, baseline adherence to the Mediterranean diet, body mass index, physical activity (quartiles), alcohol intake (<5/10 gr, moderate consumption, >25/50 g in women/men), fish intake (continuous g/d) and non-fish consumption of n-3 fatty acids (continuous g/d)

Higher toenail mercury concentration was not associated with increased cardiovascular disease risk in our nested case-control study. After adjusting for age, sex, month of toenail collection, PREDIMED center, smoking, hypertension, hypercholestaerolemia, diabetes and family history of premature CHD, we found no association (OR, 0.56; 95% CI, 0.28, 1.12; p_trend_ = 0.09), as did results after further adjusting for intervention group, baseline adherence to Mediterranean diet, body mass index (BMI), physical activity, alcohol intake, fish intake and n-3 fatty acid intake from sources other than fish (OR, 0.66; 95% CI, 0.31, 1.41; p_trend_ = 0.11) (Table 2).

When we separately analyzed the Mediterranean diet intervention groups and control (low-fat) group, we continued to observe no increase in cardiovascular disease risk for the comparison between extreme quartiles of mercury exposure in unconditional logistic regression models adjusted for age, sex and PREDIMED center (Mediterranean diet arm: OR, 0.72; 95% CI, 0.33, 1.55; *p* = 0.32; Control arm: OR, 0.40; 95% CI, 0.12, 1.34; p_trend_ = 0.13), or fully-adjusted unconditional logistic regression models (Mediterranean diet: OR, 0.74; 95% CI, 0.31, 1.75; p_trend_ = 0.67; Control: OR, 0.63; 95% CI, 0.15, 2.61; p_trend_ = 0.75) (Table 2).

### Mercury exposure, fish consumption and risk of cardiovascular disease

**Fig. 1:**
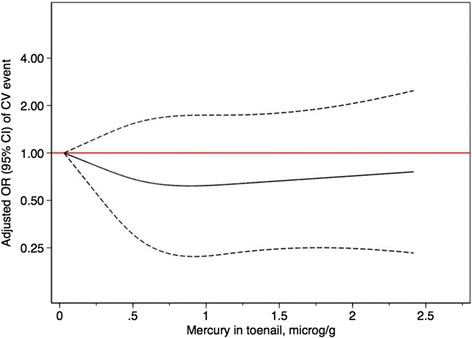
Restricted cubic spline analysis for the dose-response trend between toenail mercury levels and risk of cardiovascular disease (Odds Ratios, 95% confidence intervals from a fully-adjusted multivariable conditional logistic regression model)

Table 3 compares risk of cardiovascular disease among those with different baseline levels of both fish intake and mercury levels. Compared to those with both low mercury concentration and low fish consumption at baseline, we observed no significant associations with cardiovascular disease for any of the three other groups. Results remained null after adjusting for month of toenail collection, PREDIMED center, age, smoking, hypertension, hypercholestaerolemia, diabetes and family history of premature CHD, and after additionally adjusting for intervention group, total energy intake, BMI, physical activity, alcohol intake and n-3 fatty acid intake from non-fish sources. The point estimates in the dose-response trend suggested an initial reduction in risk with higher levels of toenail mercury that plateaued thereafter with even higher mercury levels (Fig. 1). However, the confidence intervals were compatible the null value across all mercury levels.

**Table 3:** Relative risk of cardiovascular disease, according to baseline toenail mercury and fish/seafood consumption, among case participants and matched controls in the PREDIMED trial

	Baseline toenail Mercury and fish/seafood consumption			
	Low Hg/Low Fish	Low Hg/High Fish	High Hg/Low Fish	High/High
Mean mercury (μg/g)	0.31 (0.12)	0.36 (0.09)	0.92 (0.52)	0.99 (0.56)
Fish/seafood consumption (g/d)	62 (39)	119 (39.4)	75 (36)	115 (35)
Total sample				
Cases/matched controls	41/73	37/52	29/58	39/88
Matched OR^a^ (95% CI)	1 (ref.)	1.09 (0.56, 2.11)	0.66 (0.33, 1.31)	0.85 (0.48, 1.50)
Matched OR^b^ (95% CI)	1 (ref.)	1.08 (0.53, 2.19)	0.68 (0.32, 1.44)	0.85 (0.46, 1.55)
Matched OR^c^ (95% CI)	1 (ref.)	1.18 (0.51, 2.77)	0.73 (0.34, 1.59)	0.91 (0.43, 1.93)

^a^Models are from conditional logistic regression analyses with matching factors sex and age and adjusted for PREDIMED center

^b^Additionally adjusted for month of toenail collection, smoking, hypertension, hypercholestaerolemia, diabetes and family history of premature coronary heart disease

^c^Additionally adjusted for intervention group, total energy intake, body mass index, physical activity (quartiles), alcohol intake (<5/10 gr, consumption, >25/50 g in women/men) and non-fish consumption of n-3 fatty acids (continuous g/d)

Compared to those with low mercury concentration and no increase in fish consumption from baseline to the third year of follow-up, increased fish consumption was not significantly associated with cardiovascular disease risk. Results remained null in the multivariable adjusted models. The fully-adjusted OR for the highest mercury concentration and no increase in fish consumption was 0.54; 95%CI, 0.20–1.44) (Table 4).

**Table 4:** Relative risk of cardiovascular disease, according to increases in fish/seafood consumption from baseline to third year of follow-up and baseline toenail mercury, among case participants and matched controls in the PREDIMED trial

	Increase in Fish/seafood consumption & baseline toenail Mercury			
	Low Hg/No increase in fish	Low Hg/Increased Fish	High Hg/No increase in fish	High Hg/Increased Fish
Mean mercury (μg/g)	0.33 (0.11)	0.33 (0.10)	0.99 (0.59)	0.84 (0.26)
% with high fish in MeDiet score	52	100	67	100
Total sample				
Cases/matched controls	62/87	16/38	54/116	14/30
Matched OR^a^ (95% CI)	1 (ref.)	0.46 (0.21, 1.01)	0.62 (0.38, 1.03)	0.48 (0.20, 1.15)
Matched OR^b^ (95% CI)	1 (ref.)	0.52 (0.23, 1.17)	0.65 (0.38, 1.12)	0.52 (0.20–1.34)
Matched OR^c^ (95% CI)	1 (ref.)	0.50 (0.21, 1.18)	0.66 (0.37, 1.16)	0.54 (0.20, 1.44)

^a^Models are from conditional logistic regression analyses with matching factors sex and age and adjusted for PREDIMED center

^b^Additionally djusted for month of toenail collection, smoking, hypertension, hypercholestaerolemia, diabetes and family history of premature coronary heart disease

^c^Additionally adjusted for intervention group, total energy intake, body mass index, physical activity (quartiles), alcohol intake (<5/10 gr, moderate consumption, >25/50 g in women/men) and non-fish consumption of n-3 fatty acids (continuous g/d)

### Sensitivity analyses

All sensitivity analyses of various subsets of our nested case-control study produced similar non-significant results. We observed no association after excluding cases diagnosed within one year or over five years of baseline, excluding participants with high toenail selenium levels, stratifying on baseline fish intake, restricting to participants at very high risk of cardiovascular disease, and stratifying on baseline aspirin use (Additional file 1: Tables S1–S6).

## Discussion

Our results indicate that mercury exposure, as assessed by an objective biomarker, was not associated with increased cardiovascular disease among Spanish adults age 55–80 years despite their being at high risk for cardiovascular disease and having relatively high fish consumption. There was no significant association in the total population sample used for our nested case-control study, among the subset randomized to the Mediterranean diet, or among the subset randomized to the control (low-fat) group. There was also no association between toenail mercury levels and cardiovascular disease in our total population sample when we analyzed mercury exposure in conjunction with baseline fish intake or change in fish intake between baseline and one year of follow-up.

Null results from all sensitivity analyses demonstrate that our findings are robust. Lack of association after excluding those diagnosed with cardiovascular disease more than five years after toenail collection showed that potential misclassification of exposure due to variation over time did not attenuate results. We found no association after excluding participants with high toenail selenium levels, suggesting that the potentially protective effect of selenium against mercury toxicity did not influence our associations. Similarly, we found no associations after stratifying on low versus high fish intake or adjusting for fish intake, further supporting that our lack of association between mercury exposure and cardiovascular disease risk was not attributable to the potentially protective effect of fish consumption. The lack of association after restricting to participants at very high risk of cardiovascular disease suggests that these levels of mercury exposure from moderate fish consumption probably do not represent reason for concern regarding cardiovascular risk even for the highest-risk, most susceptible individuals. We found no association after stratifying on baseline aspirin use (yes/no), suggesting that the potentially protective effect of aspirin on cardiovascular disease [45] also does not influence this relationship.

Many previous studies have investigated the relationship between mercury exposure and cardiovascular disease [11–18, 30]. The majority of findings are null, consistent with our own results. The few studies that observed positive associations had critical limitations [12, 13, 18, 19]. One case-control study that found a positive association with nonfatal myocardial infarction was retrospective and had higher rates of participation among cases. Thus, selection and recall bias may have affected their results [12]. A prospective study found a positive association with incident coronary disease, but with no dose-response relationship, and no association with overall cardiovascular mortality [13]. Two cross-sectional studies found positive associations – one with cardiovascular disease [18], and another with arterial stiffness [19]. Yet cross-sectional designs cannot establish causality, and both of these studies measured blood rather than toenail mercury concentration. Blood samples are not as well time-integrated as nail samples; red blood cells typically have a 17-week turnover, compared to 26–52 weeks for toenails [46]. However, these limitations do not entirely discount their credibility; policy and dietary recommendations regarding fish intake, mercury exposure and cardiovascular disease should be made in light of the entire body of evidence and should also take into account other non-cardiovascular harmful effects of mercury toxicity.

Our findings should not impact policies surrounding mercury pollution. The relationship between very high mercury exposure and increased risk of many health complications has been consistently demonstrated in other studies. The main application of our findings should be dietary recommendations for moderate to high fish consumption as part of ideal diets for cardiovascular disease prevention.

However, our findings do suggest that existing concerns for potential cardiovascular risk surrounding mercury exposure from fish consumption are probably unwarranted. Several aspects of our study population make these null results particularly meaningful. First, all participants are already at high risk of cardiovascular disease. Thus, any increased risk from mercury would be very likely to cause cardiovascular disease events. Second, our participants consume very high levels of fish. Per capita seafood consumption in Spain is approximately twice the average for European countries [32]. The intervention group is encouraged to consume even more fish than what is typical in Spain. The mean fish intake in our study population is approximately 92.7 g/day, compared to approximately 31.6 g/day [11], 43 g/day [13], 30.4 g/day [14], and 39.3 g/day [19] in previous studies. The observed lack of increased cardiovascular risk in this population provides evidence that consuming even the highest levels of fish consumption do not increase mercury exposure to dangerous levels. The observed null findings in this population provide evidence that mercury exposure from relatively high fish consumption (e.g. 3 servings/week) is unlikely to increase risk of cardiovascular disease. Third, our population has much higher levels of mercury (0.66 μg/g) compared to three out of four of the populations that observed a positive association between mercury exposure and cardiovascular disease (0.26 μg/g [12], 0.45 μg/g [18], and 0.30 μg/g [19]). This suggests that higher mercury exposure is not associated with increased risk of cardiovascular disease in the context of a healthy Mediterranean diet with high fish intake.

These null results are likely generalizable to populations with lower cardiovascular disease risk and lower fish consumption, since any harmful effects of mercury exposure would have even less impact among these people. Toenail mercury concentration is an objective, stable and time-integrated biomarker. Our prospective study design and our exposure assessment using an objective biomarker prevents recall bias.

In a recent report we showed the benefits of long-chain w-3 fatty acids from fish in the total data base of the trial.^47^ The hazard ratios for intake of corresponding to the levels of long-chain n-3 polyunsaturated fatty acids recommended by the International Society for the Study of Fatty Acids and Lipids were 0.84 (95% CI 0.67–1.05) for all-cause mortality, 0.61 (95% CI 0.39–0.96) for fatal cardiovascular disease, 0.54 (95% CI 0.29–0.99) for fatal coronary heart disease, and 0.49 (95% CI 0.22–1.01) for sudden cardiac death. In addition, there is extensive available evidence supporting the cardiovascular benefits of fish consumption discussed above. However, it should not be surprising that in the participants included in this sub-study we didn’t find associations between fish intake and cardiovascular disease. Virtually all established dietary guidelines recommend at least 1–2 servings of fish per week (1 serving = approximately 75 g) for cardiovascular benefits. Furthermore, evidence suggests that higher levels of fish intake does not confer added benefits.^1^ Our study population consumes an average of 95 g of fish per day. It is likely that we have too small a sample size and an insufficient range of exposure (i.e. average fish consumption is too high) to observe the cardiovascular benefits of fish consumption within this case-control substudy of mercury exposure.

There are limitations to our study that warrant continued investigation of this issue. We only have one measure of toenail mercury concentration, but toenail mercury concentrations have high reproducibility over time and low within-person to between-person variation ratio. This suggests that our single measure is a strong indicator of long-term intake [26]. However, true associations may still be attenuated. For example, it is possible that measurement error contributed to our null results. We did not have biological measurements for n-3 fatty acids. Although the FFQ has been validated in this population, fish vary in their n-3 fatty acid content [47], and the FFQ is still an imprecise measurement of n-3 fatty acids. Thus it is possible that imprecise measurement might have created residual negative confounding, as n-3 fatty acids would likely be positively associated with fish intake, and thus mercury levels, but inversely associated with cardiovascular disease. To address this, we conducted a bias analysis to assess the degree of plausible bias due to residual confounding by n-3 fatty acid intake (dichotomized at the median) based on our observed OR of 0.66 in Table 2, comparing the highest to lowest quartile of toenail mercury (Additional file 1: Table S7). We found that the odds ratio for the association between a dichotomous confounder (n-3 fatty acid) and cardiovascular disease would need to be as low as 0.2, and difference in prevalence of the confounder (high n-3 fatty acid intake in the exposed (highest quartile of mercury intake) compared to unexposed (lowest quartile of mercury intake) must be very extreme (e.g. 0.2 in exposed, 0.8 in unexposed) to render the association as detrimental. Acknowledging that toenail mercury concentration is also a biomarker for fish intake, and fish intake is associated with reduced cardiovascular disease risk, there could be negative residual confounding by fish intake or n-3 PUFA intake. This previously observed inverse association between fish intake and cardiovascular disease was not apparent in our data, probably because even in the lowest categories of fish consumption in our participants, the amount of fish consumed was already sufficiently high. While we believe it is unlikely that fish intake may strongly confound our results, because we already adjusted for fish intake, we acknowledge that residual confounding may yet exist and that our bias analysis results are subject to different interpretations. Based on previous evidence, we believe that it is unlikely, that the cardiovascular benefits of n-3 PUFAs are strong enough to account for a residual confounding of such magnitude as to explain the lack of association observed between mercury levels and cardiovascular disease. High n-3 PUFA consumption would need to be associated with approximately 0.11 the odds of cardiovascular disease compared to low n-3 PUFA consumption assuming a high association (OR = 5) between mercury and n-3 intake (Additional file 1: Table S7). A 2011 review included summarized 15 different meta-analysis RCTs and prospective cohort studies analyzing n3 PUFAs and various cardiovascular disease outcomes, including cardiovascular death, coronary heart disease, stroke and others. The lowest RR of these 15 meta-analyses was 0.62 (0.46–0.82) [48]. Despite this, we still manipulated the prevalence of the confounder to be 0.11 and higher (0.23, 0.43, 0.62, 0.81). Even at a confounder-disease OR of 0.43, which is likely still too low given that the higher RRs from the extensive meta-analyses [48], the corrected RR would be 0.90. Under the 0.23 confounder-disease OR, almost certainly too extreme, it would be 1.13. While we believe that these results indicate that residual confounding from n-3 PUFA is probably insufficient to explain our results, we admit that this interpretation may be subjective and that under some of the scenarios shown in Additional file 1: Table S7, the association mercury-CVD could be detrimental.

To minimize participant burden, our FFQ did not ask about consumption of specific types of fish. Thus, we could not obtain direct measurements of association between type of fish intake and toenail mercury concentrations. Seafood varies in mercury content [49], although mercury content of popular Spanish seafood have relatively consistent mercury levels [50]. Lastly, our sample size is small and many models are adjusted for many covariates, thus limiting our power, particularly when we adjust rather than match for sex and age in unconditional logistic regression models. However, it has been empirically demonstrated that the the rule of thumb usually suggested (adjusting for one confounder for every 10 events) can be relaxed [51]. However, the unique characteristics of our population described above, and the inverse association suggested by our point estimates, make it unlikely that we would have missed a material positive association between mercury exposure and cardiovascular disease. The role of other heavy metals in cardiovascular disease in this population merits further studies.

## Conclusion

Mercury exposure from fish intake did not appear to increase cardiovascular disease risk in our sample of high risk participants exposed to high levels of fish consumption and consequently mercury concentrations, though under some assumptions on residual confounding by n-3 fatty acids, a slightly increased risk could persist. We did not observe any substantial risk in this unique setting of elderly participants with high cardiovascular disease risk, high fish intake and relatively high toenail mercury concentrations. These findings have important public health implications, as they suggest that the mercury content in fish is not very likely to discount the well-known cardiovascular benefits of fish consumption. Based on our findings, dietary recommendations should continue to include high fish intake as part of a healthy diet. Our findings, coupled with previous extensive evidence, support the dietary recommendation of at least 1–2 servings of fish per week for the prevention of cardiovascular disease.

## Additional file

Additional file 1: Table S1. Relative Risk of Cardiovascular Disease, according to quartiles of toenail mercury, among case participants and matched controls in the PREDIMED trial, restricted to those who were not diagnosed with cardiovascular disease within one year of follow-up. **Table S2.** Relative Risk of Cardiovascular Disease, according to quartiles of toenail mercury, among case participants and matched controls in the PREDIMED trial, restricted to those who were diagnosed with cardiovascular disease within 5 years of toenail collection. **Table S3.** Relative Risk of Cardiovascular Disease, according to quartiles of toenail mercury, among case participants and matched controls in the PREDIMED trial, restricted to participants who were below the 90th percentile of toenail selenium. **Table S4.** Relative Risk of Cardiovascular Disease, according to quartiles of toenail mercury, among case participants and matched controls in the PREDIMED trial, stratified by baseline median fish intake (below/above the median). **Table S5.** Relative Risk of Cardiovascular Disease, according to quartiles of toenail mercury, among case participants and matched controls in the PREDIMED trial, restricted to participants who were at very high risk of cardiovascular disease. **Table S6.** Relative Risk of Cardiovascular Disease, according to quartiles of toenail mercury, among case participants and matched controls in the PREDIMED trial, stratified by baseline aspirin use (yes/no). **Table S7.** Bias analysis (simulation study). Sensitivity analysis to assess the degree of plausible bias due to residual confounding, assuming an unknown binary confounder (n-3 PUFA consumption; dichotomized at median) with different prevalence among exposed (highest quartile of mercury) and unexposed (lowest quartile of mercury) controls and with a relatively strong association (even after adjusting for the known and measured confounders) with cardiovascular disease. This correction was applied to our observed OR=0.66 for the highest versus lowest quartile of mercury exposure. (DOC 153 kb)

## Abbreviations

BMI: Body mass index
CHD: Coronary heart disease
DHA: Docosahexaenoic acid
EPA: Eicosapentaenoic acid
INAA: Instrumental neutron activation analysis
n-3: Omega-3
OR: Odds ratio
PREDIMED: PREvención con DIeta MEDiterránea (Prevention with Mediterranean diet)
PUFA: Poly-unsaturated fatty acids
