# Supplementary material for: Mercury exposure and risk of cardiovascular disease: a nested case-control study in the PREDIMED (PREvention with MEDiterranean Diet) study
Source: BMC Cardiovasc Disord. 2017 Jan 5;17:9. doi: 10.1186/s12872-016-0435-8 (PMC5216562; doi:10.1186/s12872-016-0435-8)
Supplement: Additional file 1: Table S1. — Relative Risk of Cardiovascular Disease, according to quartiles of toenail mercury, among case participants and matched controls in the PREDIMED trial, restricted to those who were not diagnosed with cardiovascular disease within one year of follow-up. Table S2. Relative Risk of Cardiovascular Disease, according to quartiles of toenail mercury, among case participants and matched controls in the PREDIMED trial, restricted to those who were diagnosed with cardiovascular disease within 5 years of toenail collection. Table S3. Relative Risk of Cardiovascular Disease, according to quartiles of toenail mercury, among case participants and matched controls in the PREDIMED trial, restricted to participants who were below the 90th percentile of toenail selenium. Table S4. Relative Risk of Cardiovascular Disease, according to quartiles of toenail mercury, among case participants and matched controls in the PREDIMED trial, stratified by baseline median fish intake (below/above the median). Table S5. Relative Risk of Cardiovascular Disease, according to quartiles of toenail mercury, among case participants and matched controls in the PREDIMED trial, restricted to participants who were at very high risk of cardiovascular disease. Table S6. Relative Risk of Cardiovascular Disease, according to quartiles of toenail mercury, among case participants and matched controls in the PREDIMED trial, stratified by baseline aspirin use (yes/no). Table S7. Bias analysis (simulation study). Sensitivity analysis to assess the degree of plausible bias due to residual confounding, assuming an unknown binary confounder (n-3 PUFA consumption; dichotomized at median) with different prevalence among exposed (highest quartile of mercury) and unexposed (lowest quartile of mercury) controls and with a relatively strong association (even after adjusting for the known and measured confounders) with cardiovascular disease. This correction was applied to our observed OR=0.66 for the highest versus lowest q [file 12872_2016_435_MOESM1_ESM.doc]

**Table S1** Relative Risk of Cardiovascular Disease, according to quartiles of toenail mercury, among case participants and matched controls in the PREDIMED trial, restricted to those who were not diagnosed with cardiovascular disease within one year of follow-up.

| Variable |  | Sex-specific quartiles of toenail mercury | | | |  | |
| --- | --- | --- | --- | --- | --- | --- | --- |
|  | number of cases | Q1 | Q2 | Q3 | Q4 | | p for trend |
|  |  |  |  |  |  | |  |
| Mean mercury (μg/g) |  | 0.25 (0.07) | 0.43 (0.05) | 0.65 (0.10) | 1.30 (0.62) | |  |
|  |  |  |  |  |  | |  |
| **Restricted Sample** |  |  |  |  |  | |  |
| Cases / controls | 124/271 | 27/72 | 28/72 | 33/66 | 36/61 | |  |
| Adjusted OR^1^ (95% CI) |  | 1 (ref.) | 1.01 (0.50, 1.72) | 0.69 (0.37, 1.29) | 0.58 (0.30, 1.12) | | 0.08 |
| Adjusted OR^2^ (95% CI) |  | 1 (ref.) | 0.94 (0.49, 1.77) | 0.72 (0.38, 1.38) | 0.57 (0.29, 1.13) | | 0.08 |
| Adjusted OR^3^ (95% CI) |  | 1 (ref.) | 1.01 (0.52, 1.96) | 0.75 (0.38, 1.46) | 0.68 (0.34, 1.38) | | 0.21 |

^1^ Models are from multivariate-adjusted logistic regression, adjusted for sex, age, and PREDIMED center.

^2^ Additionally adjusted for month of toenail collection, smoking, hypertension, hypercholestaerolemia, diabetes and family history of premature coronary heart disease.

^3^ Additionally adjusted for intervention group, baseline adherence to the Mediterranean diet, body mass index, physical activity (quartiles), alcohol intake (<5/10 gr, moderate consumption, >25/50 g in women/men), fish intake (continuous g/d), and non-fish consumption of n-3 fatty acids (continuous g/d).

**Table S2** Relative Risk of Cardiovascular Disease, according to quartiles of toenail mercury, among case participants and matched controls in the PREDIMED trial, restricted to those who were diagnosed with cardiovascular disease within 5 years of toenail collection

| Variable |  | Sex-specific quartiles of toenail mercury | | | |  | |
| --- | --- | --- | --- | --- | --- | --- | --- |
|  | number of cases | Q1 | Q2 | Q3 | Q4 | | p for trend |
|  |  |  |  |  |  | |  |
| Mean mercury (μg/g) |  | 0.25 (0.08) | 0.43 (0.05) | 0.65 (0.10) | 1.28 (0.57) | |  |
|  |  |  |  |  |  | |  |
| **Restricted Sample** |  |  |  |  |  | |  |
| Cases / controls | 120/271 | 36/61 | 31/66 | 26/72 | 27/72 | |  |
| Adjusted OR^1^ (95% CI) |  | 1 (ref.) | 0.88 (0.48, 1.65) | 0.59 (0.31, 1.12) | 0.56 (0.29, 1.08) | | 0.06 |
| Adjusted OR^2^ (95% CI) |  | 1 (ref.) | 0.91 (0.48, 1.73) | 0.59 (0.31, 1.16) | 0.55 (0.28, 1.08) | | 0.05 |
| Adjusted OR^3^ (95% CI) |  | 1 (ref.) | 1.00 (0.51, 1.97) | 0.65 (0.32, 1.29) | 0.71 (0.35, 1.45) | | 0.24 |

^1^ Models are from multivariate-adjusted logistic regression, adjusted for sex, age, and PREDIMED center.

^2^ Additionally adjusted for month of toenail collection, smoking, hypertension, hypercholestaerolemia, diabetes and family history of premature coronary heart disease.

^3^ Additionally adjusted for intervention group, baseline adherence to the Mediterranean diet, body mass index, physical activity (quartiles), alcohol intake (<5/10 gr, moderate consumption, >25/50 g in women/men), fish intake (continuous g/d), and non-fish consumption of n-3 fatty acids (continuous g/d).

**Table S3** Relative Risk of Cardiovascular Disease, according to quartiles of toenail mercury, among case participants and matched controls in the PREDIMED trial, restricted to participants who were below the 90^th^ percentile of toenail selenium.

| Variable |  | Sex-specific quartiles of toenail mercury | | | |  | |
| --- | --- | --- | --- | --- | --- | --- | --- |
|  | number of cases | Q1 | Q2 | Q3 | Q4 | | p for trend |
|  |  |  |  |  |  | |  |
| Mean mercury (μg/g) |  | 0.24 (0.08) | 0.43 (0.05) | 0.65 (0.10) | 1.32 (0.65) | |  |
| Mean selenium (µg/kg) |  | 0.52 (0.06) | 0.53 (0.07) | 0.55 (0.77) | 0.56 (0.06) | |  |
|  |  |  |  |  |  | |  |
| **Restricted Sample** |  |  |  |  |  | |  |
| Cases / controls | 125/242 | 42/57 | 37/59 | 31/64 | 25/62 | |  |
| Adjusted OR^1^ (95% CI) |  | 1 (ref.) | 0.91 (0.49, 1.67) | 0.62 (0.33, 1.15) | 0.48 (0.24, 0.93) | | 0.02 |
| Adjusted OR^2^ (95% CI) |  | 1 (ref.) | 0.95 (0.51, 1.78) | 0.67 (0.35, 1.27) | 0.49 (0.24, 0.97) | | 0.02 |
| Adjusted OR^3^ (95% CI) |  | 1 (ref.) | 1.16 (0.59, 2.28) | 0.78 (0.40, 1.53) | 0.67 (0.32, 1.40) | | 0.18 |

^1^ Models are from multivariate-adjusted logistic regression, adjusted for sex, age, and PREDIMED center.

^2^ Additionally adjusted for month of toenail collection, smoking, hypertension, hypercholestaerolemia, diabetes and family history of premature coronary heart disease.

^3^ Additionally adjusted for intervention group, baseline adherence to the Mediterranean diet, body mass index, physical activity (quartiles), alcohol intake (<5/10 gr, moderate consumption, >25/50 g in men/women), fish intake (continuous g/d), and non-fish consumption of n-3 fatty acids (continuous g/d).

**Table S4** Relative Risk of Cardiovascular Disease, according to quartiles of toenail mercury, among case participants and matched controls in the PREDIMED trial, stratified by baseline median fish intake (below/above the median)

| Variable |  | Sex-specific quartiles of toenail mercury | | | |  | |
| --- | --- | --- | --- | --- | --- | --- | --- |
|  | number of cases | Q1 | Q2 | Q3 | Q4 | | p for trend |
|  |  |  |  |  |  | |  |
| Mean mercury (μg/g) |  | 0.25 (0.08) | 0.43 (0.05) | 0.65 (0.10) | 1.30 (0.62) | |  |
|  |  |  |  |  |  | |  |
| **Low Fish Intake** |  |  |  |  |  | |  |
| Cases / controls | 70/131 | 25/41 | 17/31 | 17/34 | 11/25 | |  |
| Adjusted OR^1^ (95% CI) |  | 1 (ref.) | 0.98 (0.42, 2.26) | 0.70 (0.30, 1.62) | 0.41 (0.14, 1.18) | | 0.08 |
| Adjusted OR^2^ (95% CI) |  | 1 (ref.) | 1.21 (0.48, 3.03) | 0.72 (0.29, 1.76) | 0.35 (0.11, 1.10) | | 0.05 |
| Adjusted OR^3^ (95% CI) |  | 1 (ref.) | 1.45 (0.54, 3.93) | 0.89 (0.33, 2.40) | 0.46 (0.13, 1.59) | | 0.54 |
|  |  |  |  |  |  | |  |
| **High Fish Intake** |  |  |  |  |  | |  |
| Cases / controls | 76/140 | 19/20 | 21/35 | 16/38 | 20/47 | |  |
| Adjusted OR^1^ (95% CI) |  | 1 (ref.) | 0.68 (0.27, 1.68) | 0.49 (0.20, 1.24) | 0.46 (0.19, 1.13) | | 0.11 |
| Adjusted OR^5^ (95% CI) |  | 1 (ref.) | 0.70 (0.27, 1.83) | 0.56 (0.21, 1.52) | 0.47 (0.18, 1.24) | | 0.15 |
| Adjusted OR^6^ (95% CI) |  | 1 (ref.) | 0.75 (0.26, 2.10) | 0.51 (0.17, 1.49) | 0.57 (0.20, 1.61) | | 0.58 |
|  |  |  |  |  |  | |  |

^1^ Models are from multivariate-adjusted logistic regression, adjusted for sex, age, and PREDIMED center.

^2^ Additionally adjusted for month of toenail collection, smoking, hypertension, hypercholestaerolemia, diabetes and family history of premature coronary heart disease.

^3^ Additionally adjusted for intervention group, baseline adherence to the Mediterranean diet, body mass index, physical activity (quartiles), alcohol intake (<5/10 gr, moderate consumption, >25/50 g in women/men), fish intake (continuous g/d), and non-fish consumption of n-3 fatty acids (continuous g/d).

**Table S5** Relative Risk of Cardiovascular Disease, according to quartiles of toenail mercury, among case participants and matched controls in the PREDIMED trial, restricted to participants who were at very high risk of cardiovascular disease^1^.

| Variable |  | Sex-specific quartiles of toenail mercury | | | |  | |
| --- | --- | --- | --- | --- | --- | --- | --- |
|  | number of cases | Q1 | Q2 | Q3 | Q4 | | p for trend |
|  |  |  |  |  |  | |  |
| Mean mercury (μg/g) |  | 0.23 (0.08) | 0.41 (0.04) | 0.66 (0.11) | 1.27 (0.83) | |  |
|  |  |  |  |  |  | |  |
| **Restricted Sample** |  |  |  |  |  | |  |
| Cases / controls | 33/47 | 9/13 | 7/9 | 8/11 | 9/14 | |  |
| Adjusted OR^2^ (95% CI) |  | 1 (ref.) | 1.05 (0.25, 4.35) | 0.97 (0.26, 3.64) | 1.00 (0.27, 3.60) | | 0.97 |
| Adjusted OR^3^ (95% CI) |  | 1 (ref.) | 1.50 (0.27, 8.43) | 1.46 (0.29, 7.41) | 1.53 (0.30, 7.86) | | 0.67 |
| Adjusted OR^4^ (95% CI) |  | 1 (ref.) | 3.50 (0.37, 32.85) | 2.83 (0.35, 22.55) | 2.24 (0.25, 19.91) | | 0.74 |

^1^ Participants were older than age 70 and were diagnosed with both diabetes and hypertension before baseline.

^2^ Models are from multivariate-adjusted logistic regression, adjusted for sex, age, and PREDIMED center.

^3^ Additionally adjusted for month of toenail collection, smoking, hypercholestaerolemia, and family history of premature coronary heart disease.

^4^ Additionally adjusted for intervention group, baseline adherence to the Mediterranean diet, body mass index, physical activity (quartiles), alcohol intake (<5/10 gr, moderate consumption, >25/50 g in women/men), fish intake (continuous g/d), and non-fish consumption of n-3 fatty acids (continuous g/d).

**Table S6** Relative Risk of Cardiovascular Disease, according to quartiles of toenail mercury, among case participants and matched controls in the PREDIMED trial, stratified by baseline aspirin use (yes/no)

| Variable |  | Sex-specific quartiles of toenail mercury | | | |  | |
| --- | --- | --- | --- | --- | --- | --- | --- |
|  | number of cases | Q1 | Q2 | Q3 | Q4 | | p for trend |
|  |  |  |  |  |  | |  |
| Mean mercury (μg/g) |  | 0.25 (0.08) | 0.43 (0.05) | 0.65 (0.10) | 1.30 (0.62) | |  |
|  |  |  |  |  |  | |  |
| **No Aspirin Use** |  |  |  |  |  | |  |
| Cases / controls | 106/210 | 32/42 | 28/57 | 22/55 | 24/56 | |  |
| Adjusted OR^1^ (95% CI) |  | 1 (ref.) | 0.71 (0.36, 1.42) | 0.47 (0.23, 0.98) | 0.51 (0.25, 1.05) | | 0.07 |
| Adjusted OR^2^ (95% CI) |  | 1 (ref.) | 0.65 (0.32, 1.35) | 0.48 (0.22, 1.02) | 0.51 (0.24, 1.08) | | 0.10 |
| Adjusted OR^3^ (95% CI) |  | 1 (ref.) | 0.72 (0.33, 1.57) | 0.56 (0.25, 1.26) | 0.65 (0.29, 1.46) | | 0.36 |
|  |  |  |  |  |  | |  |
| **Aspirin Use** |  |  |  |  |  | |  |
| Cases / controls | 41/61 | 12/19 | 11/9 | 11/17 | 7/16 | |  |
| Adjusted OR^1^ (95% CI) |  | 1 (ref.) | 2.82 (0.78, 10.22) | 0.81 (0.24, 2.71) | 0.81 (0.22, 2.92) | | 0.37 |
| Adjusted OR^5^ (95% CI) |  | 1 (ref.) | 2.97 (0.67, 13.18) | 0.78 (0.20, 3.00) | 0.75 (0.17, 3.25) | | 0.35 |
| Adjusted OR^6^ (95% CI) |  | 1 (ref.) | 5.12 (0.95, 27.66) | 1.27 (0.27, 6.06) | 0.78 (0.16, 3.77) | | 0.44 |
|  |  |  |  |  |  | |  |

^1^ Models are from multivariate-adjusted logistic regression, adjusted for sex, age, and PREDIMED center.

^2^ Additionally adjusted for month of toenail collection, smoking, hypertension, hypercholestaerolemia, diabetes and family history of premature coronary heart disease.

^3^ Additionally adjusted for intervention group, baseline adherence to the Mediterranean diet, body mass index, physical activity (quartiles), alcohol intake (<5/10 gr, moderate consumption, >25/50 g in women/men), fish intake (continuous g/d), and non-fish consumption of n-3 fatty acids (continuous g/d).

**Table S7** Bias analysis (simulation study). Sensitivity analysis to assess the degree of plausible bias due to residual confounding, assuming an unknown binary confounder (n-3 PUFA consumption; dichotomized at median) with different prevalence among exposed (highest quartile of mercury) and unexposed (lowest quartile of mercury) controls and with a relatively strong association (even after adjusting for the known and measured confounders) with cardiovascular disease. This correction was applied to our observed OR=0.66 for the highest versus lowest quartile of mercury exposure.

| **Prevalence of confounder (high n-3 PUFA) in Exposed (highest quartile of mercury)** | **Prevalence of confounder (high n-3 PUFA) in unexposed (lowest quartile of mercury)** | **OR confounder (n-3 PUFA)-disease (cardiovascular disease)** | **OR confounder (n-3 PUFA)-exposure (mercury)** | **Residual bias correction** | **Externally Adjusted OR** |
| --- | --- | --- | --- | --- | --- |
| 0.80 | 0.44 | 0.11 | 5.00 | 0.48 | 1.39 |
| 0.80 | 0.44 | 0.23 | 5.00 | 0.58 | 1.13 |
| 0.80 | 0.44 | 0.43 | 5.00 | 0.73 | 0.91 |
| 0.80 | 0.44 | 0.62 | 5.00 | 0.84 | 0.79 |
| 0.80 | 0.44 | 0.81 | 5.00 | 0.93 | 0.71 |
|  |  |  |  |  |  |
| 0.80 | 0.50 | 0.20 | 4.00 | 0.60 | 1.10 |
| 0.80 | 0.50 | 0.40 | 4.00 | 0.74 | 0.89 |
| 0.80 | 0.50 | 0.60 | 4.00 | 0.85 | 0.78 |
| 0.80 | 0.50 | 0.80 | 4.00 | 0.93 | 0.71 |
|  |  |  |  |  |  |
| 0.80 | 0.20 | 0.20 | 16.00 | 0.43 | 1.54 |
| 0.80 | 0.20 | 0.40 | 16.00 | 0.59 | 1.12 |
| 0.80 | 0.20 | 0.60 | 16.00 | 0.74 | 0.89 |
| 0.80 | 0.20 | 0.80 | 16.00 | 0.88 | 0.75 |
